# Modulation of cellular redox homeostasis by the endocannabinoid system

**DOI:** 10.1098/rsob.150276

**Published:** 2016-04-27

**Authors:** Christopher Lipina, Harinder S. Hundal

**Affiliations:** Division of Cell Signalling and Immunology, Sir James Black Centre, School of Life Sciences, University of Dundee, Dundee DD1 5EH, UK

**Keywords:** endocannabinoid system, reactive oxygen species, antioxidant, redox homeostasis, oxidative stress, cannabinoid receptor

## Abstract

The endocannabinoid system (ECS) and reactive oxygen species (ROS) constitute two key cellular signalling systems that participate in the modulation of diverse cellular functions. Importantly, growing evidence suggests that cross-talk between these two prominent signalling systems acts to modulate functionality of the ECS as well as redox homeostasis in different cell types. Herein, we review and discuss evidence pertaining to ECS-induced regulation of ROS generating and scavenging mechanisms, as well as highlighting emerging work that supports redox modulation of ECS function. Functionally, the studies outlined reveal that interactions between the ECS and ROS signalling systems can be both stimulatory and inhibitory in nature, depending on cell stimulus, the source of ROS species and cell context. Importantly, such cross-talk may act to maintain cell function, whereas abnormalities in either system may propagate and undermine the stability of both systems, thereby contributing to various pathologies associated with their dysregulation.

## 1. Introduction

**Figure 1.**
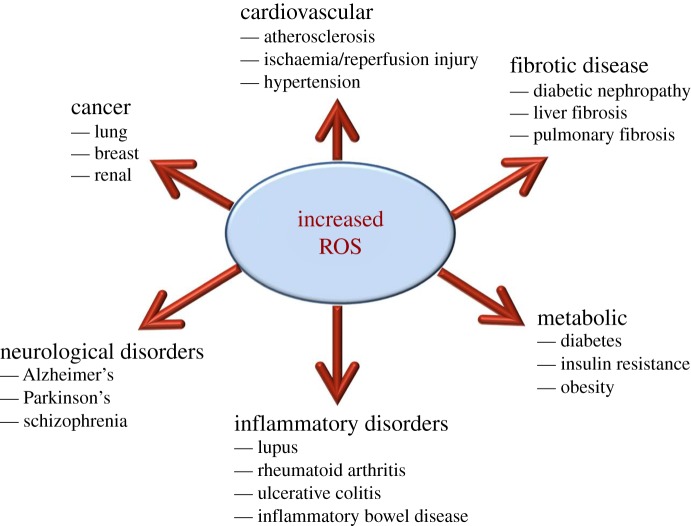
ROS involvement in disease pathogenesis. Schematic of the involvement of increased ROS production in the development of various pathologies.

The cellular redox environment constitutes a delicate balance between the production of reactive oxygen species (ROS) and their removal by antioxidant enzymes and small-molecular-weight antioxidants. At low concentrations, ROS are involved in regulating numerous physiological events, including their ability to mediate signal transduction from membrane receptors, thereby facilitating the activation of multiple proteins and enzymes [1,2]. However, excess accumulation of intracellular ROS causes oxidative stress, which can damage cellular membranes, promote mitochondrial injury and induce cell death, thereby negatively impacting upon cell function and survival [3–5]. Notably, this is largely owing to the damaging effects that free radicals convey upon cellular lipids, proteins and DNA, thus impairing their normal function. Accordingly, the dysregulation of redox homeostasis has been linked with the development of various pathologies, including those associated with metabolic disorders such as type 2 diabetes and obesity, cardiovascular disease, as well as various neurodegenerative disorders (e.g. Alzheimer's disease, Parkinson's disease and multiple sclerosis; figure 1) [6–11]. Consequently, there is growing interest in identifying cellular pathways and/or processes that can regulate ROS levels, for example by altering the balance between pro-oxidants and free radical scavenging molecules. In this review, we explore experimental evidence supporting a role for the endocannabinoid system (ECS) in the modulation of redox homeostasis and provide examples of how this relationship may impact upon cellular function.

## 2. Reactive oxygen species: generation and neutralization

**Figure 2.**
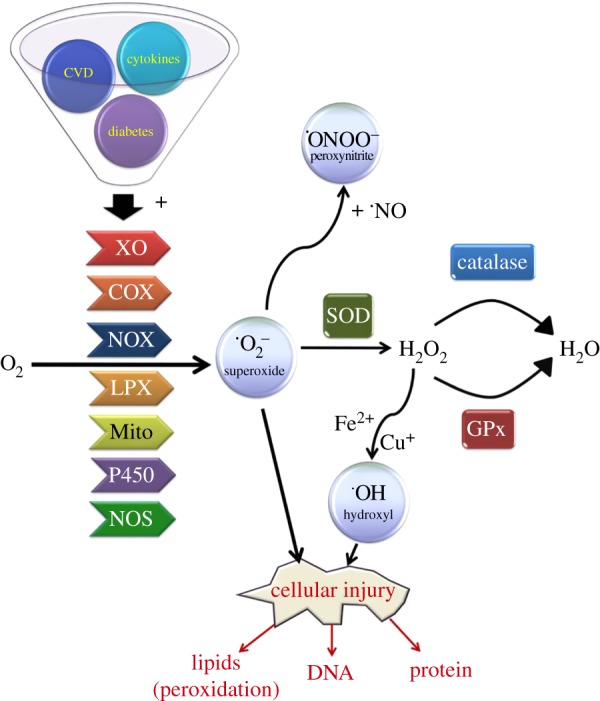
Summary of pathways involved in ROS production and clearance. Pathological conditions such as chronic inflammation, cardiovascular disease (CVD), as well as obesity and diabetes can lead to the aberrant production of various oxygen radicals from molecular oxygen, including superoxide (O_2_^−^), peroxides and hydroxyl radicals. A number of different enzymes have been implicated in mediating this process including NADPH oxidase (NOX), xanthine oxidase (XO), cyclooxygenase (COX), lipoxygenase (LPX), nitric oxide synthase (NOS) and cytochrome P450 isoforms as well as ROS derived from mitochondria (Mito). In response, cells will often initiate an antioxidant response that facilitates the neutralization of ROS into less harmful products by activating enzymes such as superoxide dismutase (SOD), catalase and glutathione peroxidase, in an attempt to alleviate the damaging effects of ROS upon lipid, protein and DNA integrity.

ROS are oxygen-containing molecules that are highly reactive in redox reactions, and are primarily produced by two metabolic sources: the mitochondrial electron-transport chain, and/or through oxygen-metabolizing enzymatic reactions such as those catalysed by xanthine oxidases, the cytochrome P450 system, NADPH oxidases, myeloperoxidase, lipoxygenase and nitric oxide synthase [12–16] (figure 2). Oxygen concentrations can also act as a key determinant of ROS production. Indeed, molecular oxygen is the terminal electron acceptor during energy production whereby it accepts an additional electron to create superoxide (**^.^**O_2_^−^), a highly reactive form of oxygen. Notably, the superoxide anion can act as a precursor for the formation of other ROS moieties including peroxynitrite (ONOO^−^), and hydroxyl radicals (**^.^**OH) through its reaction with transition metals (e.g. cuprous and ferrous ions; figure 2).

Importantly, there is substantial evidence supporting a role for ROS as key signalling intermediates that can regulate numerous cellular processes, including cell growth and proliferation, by modulating a number of different protein kinases and ion channels [10,17–20]. Cellular ROS levels are maintained by controlling the production and neutralization of ROS by various antioxidant enzymes and small-molecular-weight antioxidants. For example, superoxide is converted to hydrogen peroxide by members of the superoxide dismutase (SOD) family of enzymes, including manganese-dependent SOD (MnSOD), copper/zinc-dependent SOD (CuZnSOD) and extracellular SOD (EcSOD). MnSOD is a mitochondrial enzyme that functions to neutralize ROS generated by these organelles [21]. In contrast, CuZnSOD resides within both the cytoplasm and nucleus, while EcSOD is present in the plasma membrane and extracellular space [22]. Two other key antioxidant enzymes include catalase and glutathione peroxidase (GPx), which act to neutralize H_2_O_2_ by catalysing its conversion to water. Different isozymes of GPx are present in most subcellular compartments, and function to convert reduced monomeric glutathione (GSH; γ-l-glutamyl-l-cysteinyl-glycine) into its oxidized form (GSSG; glutathione disulfide) using hydrogen peroxide as a substrate, whereas catalase is found primarily in peroxisomes [23–25]. In the case of glutathione, its cysteine residue forms a redox-active thiol group which becomes oxidized when GSH reduces target molecules [26]. Additional intracellular small-molecular-weight antioxidants include cysteine, vitamin C (ascorbic acid) and vitamin E (α-tocopherol). Alternatively, chemical antioxidants such as *N*-acetyl-l-cysteine are also widely used as ROS scavengers.

## 3. The endocannabinoid system

The ECS is a ubiquitous ligand-directed signalling system that has been implicated in regulating a wide range of physiological processes and pathologies, including energy homeostasis, cardiovascular disease, cancer and neurodegeneration [27–30]. Two key lipid-derived molecules that act as endogenous ligands for this system are anandamide (*N*-arachidonoylethanolamine (AEA)) and 2-arachidonoylglycerol (2-AG)—commonly referred to as endocannabinoids. Both AEA and 2-AG can be synthesized on demand within the plasma membrane from arachidonic acid-derived lipids [31,32]. Anandamide generation from its membrane phospholipid precursor *N*-acylphosphatidylethanolamine (NAPE) is driven by the action of the enzyme NAPE-hydrolysing phospholipase D (NAPE-PLD) [33]. In contrast, phospholipase C-mediated cleavage of membrane phosphatidylinositols gives rise to a diacylglycerol precursor whose subsequent hydrolysis (via diacylglycerol lipase activity) permits the formation of 2-AG [34]. In addition to these synthetic pathways, enzymes that catalyse the degradation of anandamide and 2-AG have also been characterized, including fatty acid amide hydroxylase (FAAH) and monoacylglycerol lipase (MAGL), respectively [35].

Both AEA and 2-AG evoke cellular and physiological responses through binding and activating two distinct G protein-coupled receptors identified as the cannabinoid type 1 (CB1R) and type 2 (CB2R) receptors [36–39]. Indeed, various synthetic CB1R and/or CB2R agonists (e.g. CP-55,940, ACEA, WIN-55,212-2, JWH-133 and HU-210) have been used to provide mechanistic insight into the regulation of cellular processes by the ECS (table 1) [40,46,47,50,51]. Importantly, these are often applied in combination with selective receptor antagonists to determine receptor-specific responses. Such cannabinoid receptor blockers act either by competitively binding and preventing activation of a receptor by an agonist (i.e. as an antagonist), and/or function as inverse agonists through suppressing spontaneous (ligand-free) receptor signalling. For example, SR141716 (also known as rimonabant) has been shown to act as both a CB1R antagonist and an inverse agonist (table 1) [52,53]. Notably, endocannabinoids have also been reported to mediate some of their biological effects through alternative molecular targets such as the orphan G protein-coupled receptor GPR55, the transient receptor potential cation channel (TRPV1), as well as the peroxisome proliferator-activated receptor (PPAR) alpha and gamma isoforms [54–56].

**Table 1.** Synthetic modulators of cannabinoid receptor function. Citations refer to studies performed using the compounds listed in order to elucidate the functional role of CB1R and CB2R.

name	activity at CB1 (*K*_i_ in nM)	activity at CB2 (*K*_i_ in nM)	comments	references
ACEA	1.4 ± 0.3	>2000	selective CB1 receptor agonist	[40,41]
AM251	7.5	2000–3000	selective CB1 receptor antagonist/inverse agonist	[42,43]
SR141716	1.8 ± 0.2	—	selective CB1 receptor antagonist/inverse agonist	[44]
JWH-133	680	3.4	selective CB2 receptor agonist	[45]
AM630	5.2 × 10^3^	31.2	selective CB2 receptor antagonist/inverse agonist	[46]
CP-55940	0.5 ± 0.1	2.8 ± 0.4	non-selective potent CB1/2 receptor agonist	[47]
HU-210	0.1–0.7	0.2–0.5	non-selective potent CB1/2 receptor agonist	[48]
WIN-55,212-2	4.4 ± 1.3	1.2 ± 0.25	non-selective CB1/2 receptor agonist	[49]

## 4. Endocannabinoid system-mediated regulation of reactive oxygen species

There is accumulating evidence that supports a key role for the ECS in the modulation of ROS production in different cell types. For example, extensive work carried out investigating the neuroprotective properties of cannabinoid ligands has revealed a crucial link between the ECS and redox homeostasis [57–60]. For example, anandamide has been reported to attenuate neurotoxicity in response to oxidative stress [58,61]. In accord with this, the mixed CB1R/CB2R agonist WIN-55,212-2 and the plant-derived cannabinoid tetrahydrocannabinol (THC) have both been shown to protect serum-deprived astrocytes against H_2_O_2_-induced apoptosis [57]. Notably, this protective action was found to be prevented by the selective CB1R blocker SR141716, suggesting the involvement of CB1R in mediating these anti-apoptotic and/or antioxidant actions. However, it is noteworthy that the protective effect of THC may be cell specific as judged by the finding that activation of CB1R by THC increases cellular susceptibility of C6 glioblastoma cells to oxidative damage [62].

Notably, as well as responses mediated through CB1R, there is evidence to suggest that stimulation of CB2R may also convey beneficial free radical scavenging effects. Indeed, in a study by Ribeiro *et al*. [60], and co-workers, the selective CB2R agonist AM1241 was shown to almost completely block ROS generation in response to lipopolysaccharide (LPS) in BV-2 cells. Consistent with this, CB2R activation has also been reported to attenuate oxidative stress damage in various tissue types, including brain [59], kidney [63], heart [64] and liver [65]. Moreover, previous work using CB2R agonists and/or knockout mice indicates that activation of CB2R confers protection against hepatic ischaemia–reperfusion (I/R) injury, concomitant with its ability to alleviate tissue free radical damage [66–68]. Allied to this, further evidence supporting a protective role for the ECS was provided in a study by Cao *et al*. [65], who demonstrated that pharmacological inhibition of monoacylglycerol lipase, the enzyme which catalyses the hydrolysis of 2-AG, led to the suppression of oxidative stress and associated inflammation in liver tissue following hepatic I/R injury in mice [65]. Notably, the protective effects of MAGL inhibition against hepatic I/R injury involved increased endocannabinoid signalling via CB2R [65].

Conversely, stimulation of the ECS has also been demonstrated to induce the production of ROS in certain cell types [69–71]. For example, 2-AG stimulation has been shown to promote an increase in cellular ROS in BeWo trophoblasts [71]. Moreover, increased ROS and concomitant TNF-*α* cytokine production have been reported in human macrophages following CB1R activation, with both responses being attenuated by pharmacological inhibition of CB1R [69]. Moreover, CB1R inhibition using SR141716 has been found to ameliorate diabetes-induced retinal oxidative stress and inflammation, as well as improving oxidative stress in mice with non-alcoholic fatty liver disease [72]. In accord with this, evidence from a number of studies indicates that CB1R stimulation can either promote and/or facilitate oxidative stress and associated inflammation and/or cell death in human coronary artery endothelial cells [70], as well as in various models of cardiomyopathy [28,73,74], and nephropathy [75]. In addition, work by Dando *et al.* [76] showed that activation of CB1R or CB2R promotes oxidative stress in Panc1 pancreatic cancer cells resulting in the AMP-activated protein kinase (AMPK)-dependent induction of autophagy, which may, at least in part, account for the observed inhibitory effects of cannabinoid receptor ligands upon tumour cell growth [77–79]. Importantly, such findings are often supported by data demonstrating the beneficial effects on ROS-related inflammation and/or cell death following genetic deletion or pharmacological inhibition of CB1R [72,74,75,77,78].

Intriguingly, CB1R and CB2R have also been reported to differentially regulate ROS production within the same cell type. For example, a study by Han *et al.* [69] demonstrated that CB1R activation led to the upregulation of ROS levels in RAW264.7 macrophages, whereas CB2R stimulation in the same cells acted to suppress CB1R-stimulated ROS production through a pathway involving the small G protein Rap1. Therefore, modulation of these distinct cannabinoid receptors can promote differential responses with respect to cellular redox homeostasis, even within one specific cell type.

### 4.1. Mechanisms underlying cellular reactive oxygen species production by the endocannabinoid system

It is likely that the ability of the ECS to modulate the production of ROS and reactive nitrogen species is largely mediated through alterations in the expression and/or activity of enzymes implicated in the generation of these free radical species. For example, the NADPH oxidase (Nox) family of proteins are key generators of cellular ROS, particularly in central nervous system cell types such as neurons, astrocytes and microglia under pathophysiological conditions [80,81]. Notably, treatment of H_2_O_2_-stimulated HT22 neuronal cells with AEA led to the suppression of intracellular ROS and Nox2 protein/mRNA expression, with these antioxidant responses being reversed by application of the CB1R antagonist AM251 or CB1R-siRNA [82]. The authors of the same study also demonstrated that under conditions of oxidative stress, AEA acted to raise intracellular levels of SOD and GSH, while concomitantly decreasing GSSG. Importantly, these responses were prevented by AM251, indicating that AEA could restore the balance of intracellular antioxidants and pro-oxidants through targeting CB1R. In accord with these findings, treatment of streptozotocin-induced diabetic rats with Δ9-THC was also reported to increase pancreatic glutathione levels, as well as enzymatic activities of SOD and catalase [83]. Conversely, in other cell types, CB1R inhibition (by either pharmacological or genetic silencing) has been shown to attenuate ROS formation by repressing the expression of Nox isoforms [28,74,75,84]. Therefore, these findings suggest that the pathways involved in mediating the effects of cannabinoid receptor modulation upon ROS formation may be cell-type-specific. Notably, both CB1R and CB2R agonists have also been reported to repress the expression/activity of cyclooxygenase, an enzyme implicated not only in the generation of ROS but also in the degradation of anandamide [85–87].

Alternatively, the ability of ECS stimulation to regulate the production of cellular ROS may be mediated through the accumulation of toxic lipid intermediates. For example, activation of CB1R and/or CB2R has been associated with increased ceramide formation in various cell types (e.g. hepatocytes, colon cancer cells) through either increased sphingomyelin hydrolysis or ceramide de novo synthesis [88–90]. This is in accord with the reported ability of ceramide to stimulate activation of NADPH oxidase by promoting translocation of its regulatory p47^phox^ subunit to the plasma membrane [91]. Conversely, chronic CB1R stimulation has also been reported to protect against the sensitizing effects of ceramide towards H_2_O_2_-induced loss of astrocyte viability [57]. Therefore, some of the biological actions of cannabinoid receptor modulation, for example the maintenance of cell viability, may occur partly as a result of ECS modulation of ceramide and ROS formation [92].

In addition, various protein kinases may also be implicated in mediating ECS regulation of ROS. One such candidate is the cyclic AMP-dependent protein kinase A (PKA), whose regulation of ROS production has been described in several systems, including leptin-stimulated endothelial cells [93], tumour necrosis factor-treated fibrosarcoma cells [94], and in cardiomyocytes following hypoxia and reoxygenation [95]. Given the fact that PKA has been implicated in positively regulating the expression and/or activity of enzymes involved in ROS generation such as NADPH oxidase and nNOS [96,97], and that activation of CB1R can lead to reduced cellular levels of cyclic AMP and the corresponding inhibition of PKA [98], this may, at least in part, act as a means by which CB1R stimulation acts to suppress ROS formation in certain cell types, such as neuronal cells [61].

Protein kinase C (PKC) activity may also be involved in mediating the pro- and/or antioxidant responses induced by ECS stimulation. Indeed, various PKC isoforms have been shown to convey biological actions of cannabinoid ligands [99–101]. Moreover, PKC has been reported to disrupt cannabinoid actions through its ability to serine phosphorylate the CB1 receptor [102]. Based on previous findings that PKC isoforms (e.g. PKC*α* and PKCε) can facilitate and/or stimulate ROS formation, for example through activation of NADPH oxidase [103,104], it is plausible that ECS-mediated regulation of ROS homeostasis may also be mediated, at least in part, through the activity of one or more PKC isoforms, although further work will be required to determine their involvement. In addition, active PKC can stimulate the MEK/ERK1/2 signalling pathway whose activation has been shown to upregulate Nox5 activity [105], as well as being positively modulated by CB1R and/or CB2R activity [30,106–108]. Furthermore, stimulation of RAW264.7 cells by the CB1R agonist ACEA was found to induce ROS generation by a pathway dependent upon p38 MAPK, a protein kinase which can also be stimulated in response to PKC activity [69,109].

Another potential regulator of ROS production by the ECS is Rap1, a small G protein of the Ras family. In a study by Han *et al.* [69], active Rap1 was demonstrated to inhibit CB1R-induced generation of intracellular ROS and associated pro-inflammatory responses in murine peritoneal macrophages. Moreover, expression of a dominant-negative form of Rap1 profoundly enhanced CB1R-dependent ROS production. Intriguingly, the authors of the same study also demonstrated that CB2R stimulation led to the activation of Rap1, concomitant with the amelioration of CB1R-induced ROS formation in macrophages [69]. These findings therefore highlight the potential opposing effects of CB1R and CB2R activation in the modulation of ROS production in macrophages, and implicate a key role for Rap1 in regulating ROS levels by the ECS in immune cells.

In addition, the ECS has also been reported to regulate the activity of redox-sensitive transcription factors. For example, CB2R-mediated protection against myocardial infarction in mice was shown to coincide with increased nuclear translocation of the transcription factor Nrf-2 in the myocardium, concomitant with the induction of its target gene haem oxygenase-1, a key cellular antioxidant [110]. Notably, Nrf-2 functions to activate the antioxidant response element transcriptional pathway, thereby controlling the expression of genes whose protein products are involved in the detoxification and elimination of reactive oxygen intermediates [111].

Alternatively, cannabinoid ligands may act to alter cellular ROS production through controlling the production of mitochondrial-derived ROS. For example, in hepatic stellate cells, mitochondria were found to be the predominant source of ROS generated in response to 2-AG stimulation [112]. Furthermore, a recent study by Ma *et al.* [113] demonstrated that treating hippocampal neurons and tissue with the CB1R agonist ACEA increased the expression of CB1R protein in the mitochondrial membrane. Notably, in this same study, ACEA was shown to inhibit ROS generation and attenuate Ca^2+^-induced mitochondrial injury, effects that were prevented by co-application of a cell permeant CB1R antagonist (AM251), but not following co-treatment with a cell impermeable CB1R blocker (haemopressin). Therefore, it is possible that CB1R residing within mitochondria may act to control the production of ROS by these organelles, for example, by altering the expression and/or activity of components of the mitochondrial electron-transport chain, and/or by promoting changes in mitochondrial membrane potential.

### 4.2. CB1R/CB2R-independent modulation of cellular reactive oxygen species production

As well as conveying their biological effects through activation of CB1R and/or CB2R, it is possible that endocannabinoids may also regulate ROS levels by targeting alternative receptors/ion channels such as TRPV1 or GPR55 [55,56]. Indeed, one study performed by Balenga *et al.* [114] revealed that 2-AG-induced ROS production in neutrophils was significantly diminished upon co-treatment with the GPR55 activator lysophosphatylinositol (LPI). Endocannabinoids such as AEA are also known to mediate some of their cellular responses by targeting the non-selective cation channel TRPV1, whose activation has been linked to increased ROS production [115–118]. Indeed, this may be driven, at least in part, through the ability of TRPV1 stimulation to trigger Ca^2+^ signalling which is functionally coupled to ROS generating systems, in particular mitochondrial ROS production, as well as the upregulation of Nox5 activity following its phosphorylation by CAMKII, a serine/threonine protein kinase activated in response to calcium signals [119–121]. In addition, AEA has also been reported to target the PPAR family of nuclear receptors [54], whose activation is known to induce the expression of antioxidant enzymes, including catalase and glutathione peroxidase 3 [122,123].

It should be highlighted that some cannabinoid receptor ligands may also convey more direct free radical scavenging activity. For example, analysis performed in cell-free biochemical assays has revealed that some phenolic cannabinoid compounds (e.g. Δ9-THC, cannabinol, cannabidiol, CP-55,940, HU-210 and AM-404) can act as potent lipophilic antioxidants [124]. Moreover, owing to their lipophilicity, these compounds may further affect membrane-associated and intracellular signalling mechanisms, leading to changes in the activity of membrane-bound receptor systems (e.g. neurotransmitter receptors). Therefore, such free radical scavenging activity should also be considered as a potential explanation for non-CB1R/CB2R-dependent modes of antioxidant action by cannabinoid receptor ligands.

## 5. Redox-mediated regulation of the endocannabinoid system

In addition to the effects of altering ECS activity upon cellular redox homeostasis, it should be highlighted that changes in cellular redox homeostasis can also impact upon the function of the ECS. For example, activation of NADPH oxidase isoforms Nox4 and Nox1 has been reported to mediate the upregulation of CB1R expression in mouse hepatic stellate cells during *Schistosoma J.* infection [125]. Consistent with this finding, H_2_O_2_-induced oxidative stress has been reported to increase CB1R and CB2R mRNA and protein abundance in human retinal pigment epithelial (RPE) cells, as well as downregulating expression of FAAH, the enzyme involved in the degradation of anandamide [126]. The authors of the same study also demonstrated that treatment with the CB2R agonist JWH-015 protected RPE cells from oxidative damage, suggesting that upregulation of cannabinoid receptor expression and/or endocannabinoid levels may constitute part of a counter-feedback mechanism to ameliorate the damaging effects of ROS exposure under those conditions. Furthermore, Batkai *et al.* [66] have reported elevated hepatic levels of AEA and 2-AG following I/R injury in mice. Notably, the authors of the same study also demonstrated raised levels of these endocannabinoids in hepatocytes following brief exposure to pro-oxidants (hydrogen peroxide and peroxynitrite). Therefore, these findings support an important role for ROS in modulating ECS function, for example by regulating the expression of key ECS components.

## 6. Conclusion and future perspective

**Figure 3.**
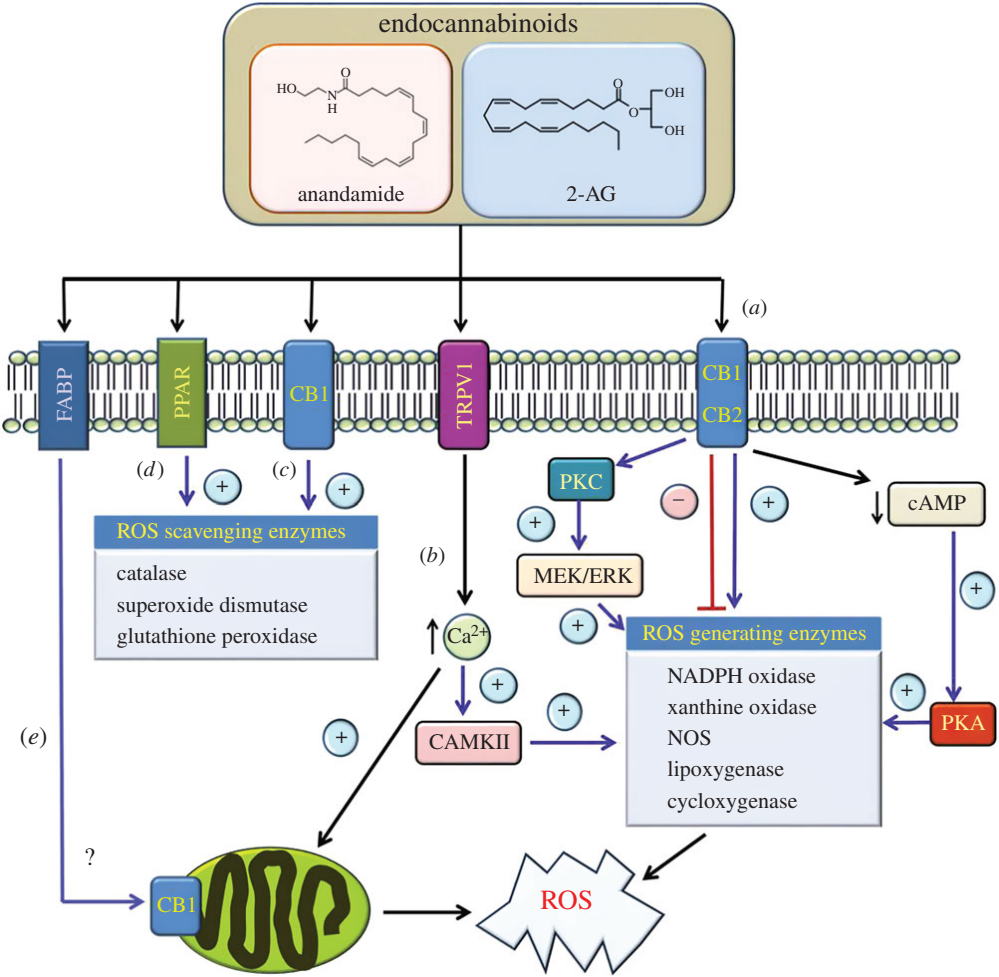
Summary of pathways implicated in the regulation of ROS production by the ECS. (*a*) Activation of CB1R and/or CB2R has been reported to either stimulate and/or repress the activity of enzymes implicated in ROS generation as indicated. For example, cannabinoid receptor activation may act to suppress cAMP-dependent activation of PKA thereby repressing the expression and/or activity of enzymes such as NADPH oxidase. Alternatively, cannabinoid receptor stimulation can activate PKC isoforms and the downstream MEK/ERK signalling axis which have been implicated in upregulating NADPH oxidase activity. (*b*) Endocannabinoids such as anandamide have also been shown to target the vanilloid receptor TRPV1. In this case, TRPV1 activation would promote an increase in intracellular calcium signalling which in turn may impact on the expression/activity of ROS generating enzymes, for example through stimulating CAMKII activity. In addition, elevated calcium levels may also promote an increase in ROS generation within mitochondria. (*c*,*d*) Endocannabinoids such as anandamide may also convey protective effects by inducing the expression of antioxidant enzymes such as catalase, superoxide dismutase and glutathione peroxidase through activation of CB1R or PPAR receptors. (*e*) Following its uptake into cells, for example via transporters such as fatty acid binding protein (FABP) isoforms, endocannabinoids such as anandamide may also directly impact upon mitochondrial ROS generation through targeting CB1R residing within these organelles.

To conclude, there is growing appreciation that the ECS may play an important role in the regulation of cellular redox homeostasis. Collectively, the evidence presented in this review indicates that ECS activation or inhibition can convey detrimental and/or beneficial biological effects through altering cellular ROS levels, depending on the cell type and/or stimulus involved. Indeed, the studies highlighted in this review show that ECS function can impact upon free radical production in a number of different ways (figure 3). Crucially, given the importance of redox status in the development of numerous pathologies, these findings identify ECS components as potential therapeutic targets for the treatment of oxidative stress-related neurological, cardiovascular and metabolic disorders.
